# The single-cell revolution in transplantation: high-resolution mapping of graft rejection, tolerance, and injury

**DOI:** 10.3389/fimmu.2025.1670683

**Published:** 2025-10-29

**Authors:** Lisha Mou, Zuhui Pu

**Affiliations:** ^1^ Shenzhen Xenotransplantation Medical Engineering Research and Development Center, Institute of Translational Medicine, The First Affiliated Hospital of Shenzhen University, Shenzhen Second People’s Hospital, Shenzhen, China; ^2^ MetaLife Lab, Shenzhen Institute of Translational Medicine, Shenzhen, China; ^3^ Imaging Department, The First Affiliated Hospital of Shenzhen University, Shenzhen Second People’s Hospital, Shenzhen, China

**Keywords:** single-cell sequencing, spatial transcriptomics, transplant immunology, allograft rejection, immune tolerance, tissue-resident memory T cells, solid organ transplantation, islet transplantation

## Abstract

Single-cell sequencing technologies are fundamentally revolutionizing our understanding of transplantation biology by providing high-resolution cellular and molecular maps of graft rejection, immune tolerance, and injury. This review systematically summarizes the application of technologies such as single-cell RNA sequencing (scRNA-seq) and spatial transcriptomics in solid organ and islet transplantation, aiming to elucidate the mechanisms that determine graft fate. Single-cell analyses have revealed profound insights unattainable by traditional methods, such as identifying key effector cell subpopulations—clonally expanded CD8+ tissue-resident memory T cells (TRM) — in acute rejection, and discovering new pathogenic pathways in chronic dysfunction, like antibody production driven by innate-like B cells. In parallel, these atlases have also uncovered the complex regulatory networks that mediate immune tolerance, composed of regulatory T cells and specific macrophage subpopulations. Furthermore, this technology has pioneered new clinical applications, including non-invasive monitoring through urinary single-cell sequencing and pre-transplant quality assessment of donor organs. By transitioning transplantation medicine from a morphology-based diagnostic model to a new era of molecular endophenotyping based on precise molecular signatures, single-cell technologies offer unprecedented opportunities for developing personalized immunosuppressive regimens, finding new therapeutic targets, and achieving non-invasive diagnostic monitoring. Although clinical translation still faces challenges, it has the potential to become a key tool for improving transplant outcomes in the future.

## 1 Introduction: beyond the microscope and bulk transcriptome

Solid organ transplantation is a revolutionary treatment for end-stage organ failure, significantly extending patient survival and improving quality of life (1, 2). However, despite substantial progress in surgical techniques, immunosuppressive protocols, and donor selection, long-term graft survival remains a challenge (3, 4). This challenge stems from an interconnected triad: allograft rejection (acute and chronic), lifelong systemic immunosuppression with its associated toxicities (e.g., nephrotoxicity, malignancy, infection), and eventual graft loss. Among these, graft rejection remains the most common and critical post-transplant complication (5–7).

Currently, the gold standard for diagnosing rejection is the pathological evaluation of graft biopsy tissue. However, this standard has inherent limitations (8–10). Firstly, histological assessment suffers from significant inter-observer variability, which affects diagnostic precision and therapeutic decision-making consistency (11). More importantly, while a pathological slide can display morphological features of cellular infiltration and tissue damage, it cannot deeply reveal the molecular and cellular mechanisms driving these pathological processes (12, 13). It fails to capture the immense heterogeneity within cell populations or to delineate the dynamic evolution of the immune response (14).

At the molecular level, the advent of bulk RNA sequencing was a significant advance, allowing researchers to quantify average gene expression levels in a tissue sample (15–17). However, this averaging of expression data is precisely its greatest drawback (18–20). A tissue sample is a complex mixture of multiple cell types, including parenchymal cells, endothelial cells, stromal cells, and various infiltrating immune cells. Bulk transcriptomics mixes the signals from these different cells, masking the contributions of rare but functionally potent key cell subpopulations (such as specific regulatory or effector immune cells) and failing to distinguish transcriptional differences between different functional states of the same cell type—differences that are critical in determining graft fate (21, 22).

The emergence of the single-cell sequencing paradigm provides an unprecedentedly powerful tool to address these limitations (23–25). The suite of technologies represented by scRNA-seq can measure the transcriptome of individual cells with unparalleled resolution (26–28). This means researchers can unbiasedly identify all cell types within a graft, precisely characterize their activation states and functional profiles, discover rare cell populations that are obscured in bulk analysis (29–31), and reconstruct the complex intercellular communication networks that drive the alloimmune response (32–35).

This technological advance is more than just an increase in resolution; it is fundamentally changing the conceptual framework of transplant immunology (36). Our perspective is shifting from a cell-type-centric model to a cell-state-centric model. In the past, we might have simply thought T cells cause rejection (37). Now, single-cell studies reveal that under the broad category of T cells, there exist multiple functionally distinct cell states, such as circulating memory T cells, tissue-resident memory T cells (TRM), exhausted T cells, and proliferating T cells (38). Crucially, these states are not static; they evolve dynamically with treatment and microenvironmental changes (39). Therefore, what determines the graft’s outcome may not be the presence or absence of a certain cell type, but rather the abundance, spatial distribution, and interaction of specific cell states (40–43). This shift redefines the core questions of transplant immunology and points the way toward developing more precise therapeutic strategies, from simply eliminating a class of cells to finely regulating specific cell states (44).

This review will systematically survey the transformative insights brought by single-cell technologies to the fields of solid organ and islet transplantation. We will dissect the cellular and molecular basis of rejection, tolerance, and injury in different organ transplants, identify universal immunological principles across organs, critically evaluate the current technical and translational challenges, and chart a roadmap toward the future of precision transplant medicine.

## 2 The high-resolution toolbox: from single cells to spatial multi-omics

To better understand the content of the following sections, this section provides a conceptual introduction to the key technologies involved in this review. Together, these technologies form a powerful toolbox that allows us to dissect the biological processes of the graft from different dimensions and at different scales.

### 2.1 Foundational technology: ScRNA-seq

ScRNA-seq is the foundation of the single-cell revolution, with its core objective being to obtain the complete messenger RNA (mRNA) information, i.e., the transcriptome, from a single cell. Its workflow typically begins with dissociating a tissue sample into a single-cell suspension. Subsequently, individual cells are physically isolated using microfluidics (e.g., the droplet method) or microwell plates. In each separate reaction unit, the cell is lysed, and its mRNA is captured by oligonucleotides with a poly(dT) sequence (45). The key innovation lies in two unique DNA barcodes on these capture sequences: a cellular barcode to identify which cell the mRNA came from, and a unique molecular identifier (UMI) to distinguish original mRNA molecules from amplification copies, thus enabling precise quantification of gene expression (46–48). The greatest advantage of scRNA-seq is that it overcomes the limitation of averaging expression values across cells, a characteristic of traditional methods, thereby revealing unprecedented cellular heterogeneity.

### 2.2 Expanding the omics universe: integrating multilayer biological information

Although scRNA-seq is powerful, it only provides one layer of biological information. To get a more complete picture, researchers have developed various multi-modal technologies (CITE-seq, scTCR/BCR-seq, scATAC-seq (49, 50), and so on) to simultaneously measure multiple omics data at the single-cell level.

CITE-seq (Cellular Indexing of Transcriptomes and Epitopes by sequencing): This is a powerful multi-modal technique that simultaneously measures mRNA (transcriptome) and cell surface proteins (immunophenotype) in the same cell by using antibodies conjugated to oligonucleotides. CITE-seq directly links a cell’s functional state (defined by gene expression) with its identifiable protein markers, providing richer and deeper cell identity information than a single omics layer (51, 52).

Single-cell T/B cell receptor sequencing (scTCR/BCR-seq): This technique, combined with scRNA-seq, allows for the simultaneous acquisition of a T or B cell’s transcriptome information and its unique antigen receptor (TCR or BCR) sequence. The sequences of TCRs and BCRs determine the antigen specificity of lymphocytes. With this technology, researchers can track the clonally expanding alloreactive lymphocytes in a graft and precisely analyze the functional phenotype and activation state of these specific clones (53, 54).

### 2.3 Spatially resolved transcriptomics: restoring positional context

A common limitation of single-cell technologies based on tissue dissociation is the loss of the cells’ spatial location information within the original tissue. The advent of spatial transcriptomics technology is aimed at providing this missing spatial information. Spatial transcriptomics technologies measure gene expression directly *in situ* on a tissue section and map this expression information back to its precise tissue coordinates (55). This allows researchers to visualize the organization of different cell types in the graft microenvironment, for example, how they interact and organize in focal areas of rejection rich in immune cells or in fibrotic niches (56).

### 2.4 The challenge of computational biology: data integration and analysis

These high-resolution technologies generate massive, high-dimensional, multi-modal datasets, and their effective analysis requires advanced computational biology workflows. Key analytical steps include cell type annotation using known marker gene databases or supervised learning classifiers (57–59); integration of data from different experiments, technology platforms, or omics layers using complex algorithms (60–63); and inference of cell-cell communication networks by analyzing the co-expression patterns of ligand-receptor pairs (64–67). To help the reader better understand these technologies, the following table (**Table 1**) summarizes their core principles and applications in the field of transplantation.

**Table 1:** Overview of single-cell and spatial technologies in transplantation.

Technology	Biological layer measured	Core principle	Key question answered in transplantation
scRNA-seq	Transcriptome	Capturing poly(A)-tailed mRNA from barcoded single cells for sequencing	What cell types are present in the graft? What are they doing (functional state)?
CITE-seq	Transcriptome + Surface Proteins	Using antibodies conjugated with DNA barcodes to simultaneously measure RNA and cell surface proteins	What is the protein phenotype of alloreactive T cells? How do function and phenotype correlate?
scTCR/BCR-seq	Transcriptome + Antigen Receptor Clonotype	Simultaneously sequencing the mRNA and the unique TCR/BCR sequence of a single T/B cell	Which T or B cell clones have expanded in the graft?
scATAC-seq	Chromatin Accessibility	Using a transposase to cut open chromatin regions, revealing active gene regulatory elements	Which gene regulatory networks are rewired to drive rejection or tolerance?
Spatial Transcriptomics	Spatially Resolved Transcriptome	Mapping gene expression information on a tissue section back to its original spatial coordinates	Where are the immune cells causing rejection located? Who are they interacting with?

## 3 Deconstructing the allograft response: organ-specific insights

This core section will systematically review the major findings from single-cell research across various organ transplant fields, highlighting the unique pathological features and immunological principles revealed in different organs.

### 3.1 Kidney: mapping the rejection atlas and pioneering non-invasive monitoring

Kidney transplantation is one of the most extensively studied areas, where the application of single-cell technology has not only greatly deepened the understanding of rejection heterogeneity but has also brought transformative new directions for clinical practice.

ScRNA-seq has provided an unprecedented high-resolution cellular map of kidney transplant rejection, with a level of detail far exceeding traditional histological classifications (68). Studies have been able to clearly distinguish different patterns of cellular infiltration and activation states in antibody-mediated rejection (ABMR), T-cell mediated rejection (TCMR), and chronic kidney transplant rejection (CKTR) (69). For example, CKTR is characterized by an increase in specific immune cell subsets (e.g., multiple subclasses of NKT cells, memory B cells) and the emergence of a newly discovered, pro-fibrotic myofibroblast population (70). ABMR, on the other hand, is closely associated with inflammatory macrophages, activated fibroblasts, and the activation of the complement signaling pathway (69, 71). Recent multi-omics integration (mRNA/miRNA plus scRNA-seq) identified six microvascular inflammation-associated miRNAs: miR-139-5p (downregulated) and miR-142-3p/150-5p/155-5p/222-3p/223-3p (upregulated), whose expression correlates with rejection severity and regulates cell-specific pathways—including endothelial MHC responses (miR-139-5p) and tubular metabolic dysfunction (miR-222-3p) (72).

A landmark discovery is that during rejection, the T cell infiltrate is actually dominated by a limited number of clonally expanded, alloreactive CD8+ T cell clones (CD8_EXP_) (39). This indicates that rejection is not a generalized process initiated by a large variety of T cells, but a highly specific attack driven by a few dominant clones. Critically, these CD8_EXP_ clones can persist in the graft for months even after successful anti-rejection therapy. They alter their phenotype, often transitioning to a TRM cell state. These persistent TRM cells form a potential pool of clonal populations that could be the source of future chronic or recurrent rejection (39). This finding fundamentally challenges our traditional notion of fully resolving rejection. Clinically, it has been observed that acute rejection, even when histologically resolved, is a major risk factor for long-term chronic rejection and graft loss (36). Single-cell research provides a powerful mechanistic explanation for this clinical phenomenon: systemic anti-rejection therapies (like steroids) may temporarily clear most circulating inflammatory cells, resulting in an improved histological appearance, but they fail to effectively eliminate the TRM cell pool deeply embedded in the tissue. These surviving TRM clones continuously release low levels of inflammatory signals, driving a slow but irreversible process of chronic fibrosis that ultimately leads to graft failure. This suggests that future therapeutic strategies must move beyond systemic immunosuppression and toward developing new therapies that can specifically target or modulate the function of this tissue-resident clonal population (73).

For chronic allograft dysfunction, the leading cause of graft loss, single-nucleus RNA sequencing (snRNA-seq) has revealed its complex cellular and molecular underpinnings. A study of kidney biopsies from patients with chronic allograft dysfunction identified two distinct states of fibrosis: a low extracellular matrix (ECM) and a high ECM state, each with unique kidney cell subclusters, immune cell types, and transcriptional profiles (74). Proximal tubular cells (PTCs) were identified as the main drivers of fibrosis, transitioning to an injured mixed tubular (MT1) phenotype that expressed markers of activated fibroblasts and myofibroblasts (74). These MT1 cells produced provisional ECM and recruited inflammatory cells, thus driving the fibrotic process. Interestingly, MT1 cells in the high ECM state showed signs of replicative repair, with evidence of dedifferentiation and nephrogenic transcriptional signatures, whereas those in the low ECM state exhibited severe metabolic dysfunction, limiting their repair potential (74). This finding shifts the paradigm of chronic rejection from a purely immune-driven process to one where the injured parenchymal cell is an active participant and driver of the pathology.

A key technological breakthrough is the demonstration that scRNA-seq of urinary sediment cells can effectively capture the immune landscape within a rejecting graft (75). During rejection, the cellular composition of the urine (e.g., increased numbers of macrophages and T cells) is highly consistent with that of the biopsy tissue. More excitingly, the key alloreactive T cell clones found in the tissue can also be detected in the urine (39). This provides a strong proof-of-concept for developing a urine-based, non-invasive liquid biopsy, which could potentially replace or supplement invasive kidney biopsies for early and dynamic monitoring of rejection.

### 3.2 Liver: unveiling tissue residency and immune privilege

Liver transplantation has attracted considerable attention due to its unique phenomenon of immune privilege, meaning liver grafts are relatively less prone to rejection and can even induce tolerance. Single-cell technologies are uncovering the cellular and molecular mechanisms behind this complex phenomenon.

Similar to the kidney, scRNA-seq studies in liver transplantation have also revealed the central driving role of CD8+ TRMs in rejection. Compared to functionally stable grafts, rejecting liver grafts have a significantly increased number of CD8+ TRMs (76). These cells exhibit a unique transcriptional signature, including high expression of genes related to activation, cytotoxicity (GZMB, IFNG), proliferation, and immune checkpoints (PD1, CTLA4) (76).

In studies of pediatric liver transplant rejection, single-cell analysis has revealed key communication pathways between immune cells. Specifically, the intercellular communication between liver-resident macrophages, Kupffer cells and expanded CD8+ T cells appears to promote T cell proliferation and persistence within the graft, thereby exacerbating rejection (53).

The findings from liver research present an interesting paradox: on one hand, the liver is considered an immune-privileged organ capable of inducing tolerance; on the other hand, single-cell studies show that when liver rejection does occur, its mechanism (driven by aggressive CD8+ TRMs) is strikingly similar to that in non-privileged organs like the kidney (76). This suggests that the liver’s immune microenvironment is not simply suppressive, but a site of highly dynamic interactions between pro- and anti-inflammatory signals. The final outcome, whether it leans toward tolerance or rejection, may depend on the balance between pro-tolerance signals (e.g., from Kupffer cells in specific states) and pro-inflammatory effector signals (e.g., from alloreactive TRMs).

### 3.3 Heart: spatially resolving the molecular anatomy of rejection

Spatial transcriptomics has revolutionized heart transplant research by systematically mapping rejection microenvironments, revealing that traditional histology masks profound molecular heterogeneity in acute cellular, antibody-mediated, and mixed-type rejection—including distinct pre-treatment signatures predictive of therapy response (77). Complementing this spatial atlas, scRNA-seq deconstructed infiltrating cell heterogeneity in rejecting grafts: endothelial cells actively upregulate MHC class II to become antigen-presenting cells that exacerbate immune attack (78), while specialized macrophage subpopulations drive regional damage (78). Critically, single-cell comparisons of tolerant versus rejecting grafts identified HIF-2α in macrophages as a master regulator of transplant tolerance, with pharmacological enhancement promoting graft acceptance in mice (79), and cell-cell communication analysis revealed CXCR3 blockade as an effective strategy to suppress rejection by disrupting chemokine-driven inflammatory circuits (80). This integrated spatial-single cell approach uncovers both pathogenic mechanisms and therapeutic opportunities with unprecedented resolution.

### 3.4 Lung: revealing immune dynamics in acute and chronic rejection

Recent advances in single-cell technologies have revolutionized our understanding of lung transplant rejection mechanisms. ScRNA-seq has uncovered profound immune cell heterogeneity in chronic lung allograft dysfunction (CLAD), particularly bronchiolitis obliterans syndrome (BOS) (81). Studies reveal that innate-like B cells differentiate into Mzb1-expressing plasma cells that locally produce IgG antibodies, directly contributing to antibody-mediated rejection—a finding validated in murine models where immunoglobulin depletion alleviated BOS severity (81). Additionally, multi-omics approaches (scRNA-seq plus scATAC-seq) demonstrate that mesenchymal cells undergo stable pro-fibrotic reprogramming, driving persistent fibrosis in rejecting lungs (82). In acute rejection, scRNA-seq of human lung biopsies shows dynamic immune shifts: cytotoxic CD8+ TRM, γδ T cells, and exhausted CD8+ T cells expand during severe acute cellular rejection, while regulatory T cells (Tregs) transiently increase in mild/recovering phases (83). Concurrently, myeloid reprogramming occurs, characterized by decreased classical monocytes/macrophages and increased TREM2+ pro-fibrotic myeloid subsets (83). Critically, these acute-phase cytotoxic T-cell expansions and myeloid reprogramming (83) may drive persistent immune activation that subsequently promotes B-cell/plasma-cell infiltration and mesenchymal fibrosis in CLAD. This suggests that targeting acute-phase immune dynamics could halt progression to chronic rejection—a clinically significant implication for intervention strategies.​

### 3.5 Islet transplantation: integrated analysis, challenges and solutions

Islet transplantation for Type 1 Diabetes faces dual challenges: inconsistent donor islet quality and post-transplant functional decline. Single-cell technologies now enable comprehensive pre-transplant evaluation through scRNA-seq, which precisely quantifies functional β-cell proportions (via INS/IAPP expression) (84) and endocrine cell composition (α-cell GCG, δ-cell SST markers) (85). Critically, scRNA-seq reveals cellular stress responses (HSP90/FOS upregulation) and functional maturity (MAFA/PDX1 expression) that predict graft viability (86). Complementing this, snRNA-seq has emerged as a transformative technology that enables analysis of cryopreserved samples while avoiding enzymatic dissociation artifacts. This approach identifies novel cellular identifiers like ZNF385D for β-cells and PTPRT for α-cells, while also resolving dynamic β-cell subpopulations, from transcriptionally active INS-pre-mRNA-rich cells to mature INS-mRNA-dominant clusters, with distinct functional capacities (86).

Mechanistically, post-transplant failure involves coordinated immune and cellular adaptation processes. Allogeneic rejection is driven by cytotoxic CD8^+^ T cells that upregulate granzyme B (GZMB) and interferon-γ (IFNG) (85), alongside proinflammatory M1 macrophages secreting CXCL9/10 chemokines (87). Simultaneously, islet cells undergo pathogenic adaptation characterized by antigen-presenting-like transformation (MHC-I/PSMB8 upregulation) (84) and β-cell dedifferentiation (loss of MAFA, gain of SOX9) (86). Cell communication networks further exacerbate rejection through CCL5-XCR1 signaling between proliferative T cells and CD8^+^ T cells (85), and TNFSF12-mediated mesenchymal-macrophage crosstalk (87). This will enable the future development of multidimensional evaluation frameworks for personalized transplantation protocols. Such frameworks could combine the advantages of snRNA-seq, like cryo-compatibility, with the comprehensive cytoplasmic transcript capture of scRNA-seq.

## 4 Universal themes across transplantation fields

By integrating the organ-specific findings above, we can identify some universal principles that have been revealed by single-cell analysis and run through the entire field of transplant immunology.

### 4.1 Cellular interactions in rejection: patterns of convergence and divergence

A common theme in kidney, liver, and heart transplantation is that clonally expanded, cytotoxic CD8+ T cells, particularly those with a TRM phenotype, are the core drivers of acute cellular rejection (73, 88). Myeloid cells (macrophages and dendritic cells) consistently appear as key mediators, maintaining the inflammatory response and T cell activation state through crosstalk with T cells (89). In contrast, the drivers of chronic rejection appear to be more organ-specific. Although T cells are still involved, scRNA-seq has uniquely revealed that in chronic lung rejection (BOS), the B cell lineage producing local antibodies plays a key role (81); whereas in the fibrotic processes of the kidney (CKTR) and lung (CLAD), pro-fibrotic mesenchymal cell subpopulations take center stage (82).

### 4.2 The signature of operational tolerance: a network-level perspective

Operational tolerance transcends singular cellular mechanisms, constituting a ​​tripartite network of peripheral, graft-intrinsic, and metabolically coordinated immunoregulation. Single-cell technologies are revealing the complex regulatory networks behind it. While the important role of Tregs in maintaining immune tolerance is well known (90), single-cell research reveals a more complex picture. Operational tolerance is not dominated by a single cell type but is the result of a multi-cellular, multi-pathway synergistic regulatory network.

Recently, scRNA-seq analysis of peripheral blood mononuclear cells has revealed the systemic immune features of operational tolerance. A pioneering study of a kidney transplant recipient who achieved operational tolerance found that their immune landscape was drastically different from that of a stable-function recipient on standard immunosuppression, and instead more closely resembled that of healthy controls (91). Specifically, the tolerant patient had higher proportions of TCL1A+ naive B cells and LSGAL1+ Tregs (91). Ligand-receptor analysis further revealed interactions between B cells and Tregs that may enhance the proliferation and suppressive function of Tregs (91). Furthermore, MSC therapy in a rat liver models dissects tolerance induction via monocyte polarization, neutrophil education (PD-L1 positive), and exhausted CD8+ T cell generation (92). This finding expands our view from mechanisms within the graft to the systemic immune system, suggesting that tolerance is an active state with systemic signatures detectable in peripheral blood, offering the possibility of identifying or tracking tolerant individuals non-invasively, a key goal in transplantation.

### 4.3 Mapping the initial insult: ischemia-reperfusion injury at cellular resolution

Ischemia-reperfusion injury (IRI) is the initial, unavoidable injury that every allograft must endure. ScRNA-seq provides a dynamic cellular map of this process. IRI is not a single event but a dynamic process, and scRNA-seq reveals how different cell types are affected over time—from the initial ischemic phase (mainly affecting cell metabolism) to the reperfusion phase (triggering a dramatic inflammatory response) (93). In studies of the kidney and liver, single-cell technology has precisely pinpointed the cell types most sensitive to IRI. In the kidney, PTCs are the main site of damage (94).

More importantly, IRI is not just a transient injury; it can sow the seeds for long-term chronic pathology. A study using snRNA-seq in a mouse model of acute kidney injury (AKI) identified a unique, pro-inflammatory and pro-fibrotic state of PTCs that fails to repair, termed failed-repair proximal tubule cells (FR-PTCs) (95). These FR-PTC cells emerge and persist after injury, express unique genes including *Vcam1*, and secrete a range of pro-inflammatory and pro-fibrotic cytokines such as *Ccl2* and *Tgfb2* (95). Pseudotemporal trajectory analysis showed that FR-PTCs represent an alternative, pathological branch diverging from the successful repair trajectory (95). This concept provides a direct mechanistic link between the initial, universal IRI and the later development of chronic fibrosis. The initial ischemic hit may induce a subset of tubular cells into this persistent pathological state, and these FR-PTC cells then drive inflammation and fibrosis through continuous signaling, ultimately leading to the chronic graft dysfunction described by McDaniels et al. (74).

To systematically summarize these findings, the following table (**Table 2**) integrates key single-cell research outcomes across different organs and clinical contexts (39, 53, 70, 75–77, 79, 81, 82, 94, 96).

**Table 2:** Key cellular and molecular findings from single-cell studies in solid organ and islet transplantation.

Organ	Clinical context	Key cell roles identified by single-cell sequencing	Key molecular pathways/markers	Ref
Kidney	Acute Cellular Rejection	Clonally expanded CD8+ T cells, especially TRM subtype	Adaptive phenotype (dependent on immunosuppressant), persistent clonal reservoir	(39)
	Chronic Rejection/Fibrosis	NKT cell subclasses, memory B cells, pro-fibrotic myofibroblasts	Increased immune cell infiltration, extracellular matrix remodeling	(70)
	Non-invasive Monitoring	Macrophages, T cells in urine (clones consistent with biopsy)	Urinary cell atlas can reflect intra-renal rejection status	(39, 75)
	Ischemia-Reperfusion Injury	Proximal tubule cells	Ferroptosis pathway, PHYH upregulation	(94)
Liver	Acute Cellular Rejection	CD8+ TRM cells	Activation, cytotoxicity, proliferation, and immune checkpoint (PD1, CTLA4) genes	(76)
	Rejection Mechanism	Interaction between Kupffer cells and CD8+ T cells	CD2-CD58 signaling pathway promotes T cell proliferation	(53)
Heart	Acute Rejection (all types)	Spatially heterogeneous cellular neighborhoods (T cells, macrophages, endothelial cells)	IFNγ/TNFα, IL6-JAK-STAT3 signaling pathway (associated with treatment response)	(77)
	Operational Tolerance	HIF-2α-expressing macrophages	HIF-2α pathway is crucial for inducing tolerance	(79)
Lung	Chronic Rejection (BOS)	Innate-like B cells and their differentiated plasma cells	Local IgG production (Mzb1, Bhlhe41)	(81)
	Chronic Rejection (CLAD)	Pro-fibrotic mesenchymal cells	Stable, pro-fibrotic transcriptional and epigenetic changes	(82)
Islet	Graft Quality Assessment	Proportion and health status of β-cells, α-cells, and other endocrine cells	Gene expression profiles related to cell stress and function	(96)
	Failure Mechanism	T lymphocytes, myeloid cells	Interaction between immune cells and islet cells, leading to graft destruction	(80, 84, 87)

## 5 From lab to clinic: challenges and future outlook

Despite the brilliant achievements of single-cell technologies in basic research, a series of technical and bioinformatic hurdles must be overcome to translate these powerful findings into routine clinical tools.

### 5.1 Technical and bioinformatic barriers to clinical application

#### 5.1.1 Sample processing issues: from biopsy to data: the artifact challenge

Dissociating solid tissue into a high-quality, viable single-cell suspension without introducing artificial transcriptomic changes is the first and a huge challenge of the entire workflow. Traditional tissue dissociation methods often require incubation with proteases (like collagenase) at 37°C, which induces transcriptional cell stress that can alter data interpretation (97). Studies have shown that 37°C collagenase digestion induces a conserved core gene set of 512 genes, including heat shock proteins and stress-response genes (like FOS and JUN) (98). These experimentally induced expression changes could be misinterpreted as true biological signals.

To address this challenge, using proteases that are active at low temperatures (cold-active proteases) for tissue dissociation has emerged as a promising solution. This method allows the entire dissociation process to be carried out at 4-6°C, a temperature at which mammalian transcriptional machinery is largely inactive, thus effectively preserving the *in vivo* gene expression patterns (99). Comparative studies have confirmed that the cold protease method can dramatically reduce gene expression artifacts, providing data that more accurately reflects the true *in vivo* biological state (99). Furthermore, the advent of snRNA-seq is another major advancement, as it can process frozen tissue, thereby bypassing the need for enzymatic digestion of fresh tissue and reducing stress artifacts (86).

#### 5.1.2 The challenge of distinguishing donor versus recipient cells

This is a fundamental challenge unique to the field of transplantation: how to distinguish which cells in a graft biopsy sample originate from the donor and which from the recipient. Without this distinction, we cannot accurately interpret the true state of immune infiltration. New bioinformatic tools to solve this problem (like scTx) have emerged, attempting to deconvolute the cell’s origin directly from scRNA-seq data using genetic variant information. However, due to issues like differing cell proportions, doublet contamination, and ambient RNA contamination, this remains a complex and yet-to-be-perfected area (100).

#### 5.1.3 Platform-specific biases and data integration

Different commercial scRNA-seq platforms (e.g., 10x Genomics Chromium, BD Rhapsody) have their own technical biases (101). They may differ in gene capture sensitivity, cell type capture efficiency, doublet rates, etc., which makes direct comparison across studies difficult. Although integrating data from scRNA-seq, scATAC-seq, CITE-seq, and spatial transcriptomics can provide extremely rich information, the computational demand is enormous (102).

#### 5.1.4 Computational bottlenecks: a guide to analysis platforms

Effective analysis of these massive, high-dimensional datasets requires complex computational workflows, and the choice of analysis platform is a key step. Currently, analysis tools can be broadly categorized into two types: code-based ecosystems and graphical user interface (GUI) programs.

Code-based Ecosystems: For researchers with programming skills, the R-based Seurat package and Bioconductor project, and the Python-based scverse ecosystem (with Scanpy at its core), offer the greatest flexibility, scalability, and reproducibility. These tools are the gold standard for academic research, supporting the full pipeline from data preprocessing to complex downstream analyses (like trajectory inference and multi-omics integration).

Graphical User Interface (GUI) Programs: For clinicians or experimental biologists lacking a programming background, a range of user-friendly GUI programs (e.g., SciDAP (103), Partek Flow, Loupe Browser, CELLxGENE, BBrowserX) increases accessibility. These platforms typically offer graphical point-and-click workflows for data visualization and standard analyses. However, their convenience often comes at the cost of flexibility, and they may not support the latest algorithms or highly customized analyses. The choice of platform depends on the research question, dataset size, and the user’s computational skills, a trade-off that needs careful consideration at the start of a project.

### 5.2 The path to clinical utility: from data to decisions

After overcoming the technical barriers, the ultimate goal is to transform this massive amount of data into useful information that can guide clinical decisions.

#### 5.2.1 Developing validated non-invasive biomarkers: urinary scRNA-seq *vs*. cell-free DNA

A key goal for clinical application is to move away from reliance on invasive biopsies. The discovery that urine cell scRNA-seq can monitor kidney rejection provides an important proof-of-concept for this (39, 75). In parallel, donor-derived cell-free DNA (dd-cfDNA) has emerged as a promising non-invasive biomarker (104). These two technologies are not in competition but are highly complementary, each providing different but equally important information.

dd-cfDNA: dd-cfDNA consists of DNA fragments released into the recipient’s blood upon the death of graft cells (105). An elevated level of dd-cfDNA in the blood is a highly sensitive but non-specific indicator of graft injury (106). It can act as a quantitative indicator, indicating that the graft is undergoing damage, but it cannot specify the cause of the damage (e.g., rejection, infection, or ischemia) (106). dd-cfDNA has a short half-life (about 30 minutes to 2 hours), making it a dynamic monitoring tool that can detect injury earlier than traditional biomarkers (105). Commercial dd-cfDNA tests are already available, making them more readily implementable in the clinic (107).

Urinary scRNA-seq: Unlike dd-cfDNA, urinary scRNA-seq provides qualitative, high-resolution information. It doesn’t just measure whether there is damage, but reveals the underlying causes and mechanisms. By analyzing the immune and kidney cells in the urine, scRNA-seq can identify specific pathogenic cell types (like clonally expanded T cells), their activation states, and the molecular pathways driving rejection. It can provide a detailed mechanistic profile of the injury rather than just a damage alert.

These two technologies can work in synergy to form a powerful future clinical monitoring paradigm. One can envision a two-step workflow: first, use the relatively low-cost, rapid-turnaround dd-cfDNA for routine, frequent screening. When dd-cfDNA levels rise significantly, this would act as an alert, triggering the second step, a more detailed and informative urinary scRNA-seq analysis. This approach could pinpoint the root cause of the injury, thereby guiding precise therapeutic interventions and potentially avoiding invasive biopsies in many cases. **Table 3** summarizes the comparison of these two non-invasive monitoring modalities.

**Table 3:** Comparison of non-invasive monitoring modalities: dd-cfDNA *vs*. urinary scRNA-seq.

Feature	Donor-derived cell-free DNA (dd-cfDNA)	Urinary scRNA-seq
Analyte	DNA fragments in circulation	Intact cells in urine
Information Provided	Quantitative: Overall level of graft injury	Qualitative: Cell identity, cell state, activation pathways, clonotype
Specificity for Rejection	Low (elevated by any cell death)	High (can identify specific alloreactive cells)
Sensitivity for Early Injury	Very High	High
Clinical Readiness	Commercially available tests	Emerging, primarily research-based
Core Advantage	Frequent, lower-cost screening tool	Detailed, mechanistic diagnostic tool
Proposed Clinical Use	Routine monitoring/screening as an alert for injury	Diagnostic follow-up to abnormal screens to guide therapy

#### 5.2.2 Personalized immunosuppression and discovering new therapeutic targets

ScRNA-seq has revealed that rejection is not a single disease, but a group of heterogeneous conditions driven by different molecular endophenotypes (77). This opens the door to precision medicine. In the future, treatment regimens could be personalized according to the cellular and molecular pathways driving a specific patient’s rejection, while attempting to reduce immunosuppressants in lower-risk patients to minimize drug toxicity (36). Furthermore, by providing an unbiased map of all active cells and pathways in a disease state, single-cell analysis is a powerful engine for drug discovery. It can not only identify entirely new therapeutic targets (such as HIF-2α, CXCR3, the B-cell pathway in BOS) but can also predict which drug targets are more likely to succeed in clinical trials by confirming their specific expression in disease-relevant tissues and cell types (79).

**Table 4** summarizes the main challenges and corresponding solutions for the clinical application of single-cell sequencing technologies (45, 68, 100–102).

**Table 4:** Challenges and solutions for the clinical translation of single-cell sequencing in transplantation.

Challenge	Impact on clinical application	Emerging solutions/future directions	Ref
Tissue Dissociation Artifacts	Reduces data reliability and reproducibility, affecting result interpretation	Standardized operating procedures; shifting to snRNA-seq for frozen tissues	(68)
Distinguishing Donor *vs*. Recipient Cells	Inability to accurately interpret immune infiltration, confounding cell origins	Development of deconvolution algorithms (e.g., scTx) that do not require extra genotyping	(100)
Reliance on Invasive Biopsies	Limits frequent monitoring, increases patient burden and risk	Development of urine- and blood-based single-cell liquid biopsy techniques	(108)
Data Complexity and Integration	Hinders the development of simple, actionable clinical metrics	Applying AI/machine learning to discover biomarkers; establishing standardized analysis pipelines	(102)
Inter-Platform Differences	Makes cross-study comparisons difficult, affecting the generalizability of results	Systematic platform benchmarking; developing computational methods to correct for platform biases	(109)

## 6 Conclusion: toward a new era of precision transplant medicine

**Figure 1:**
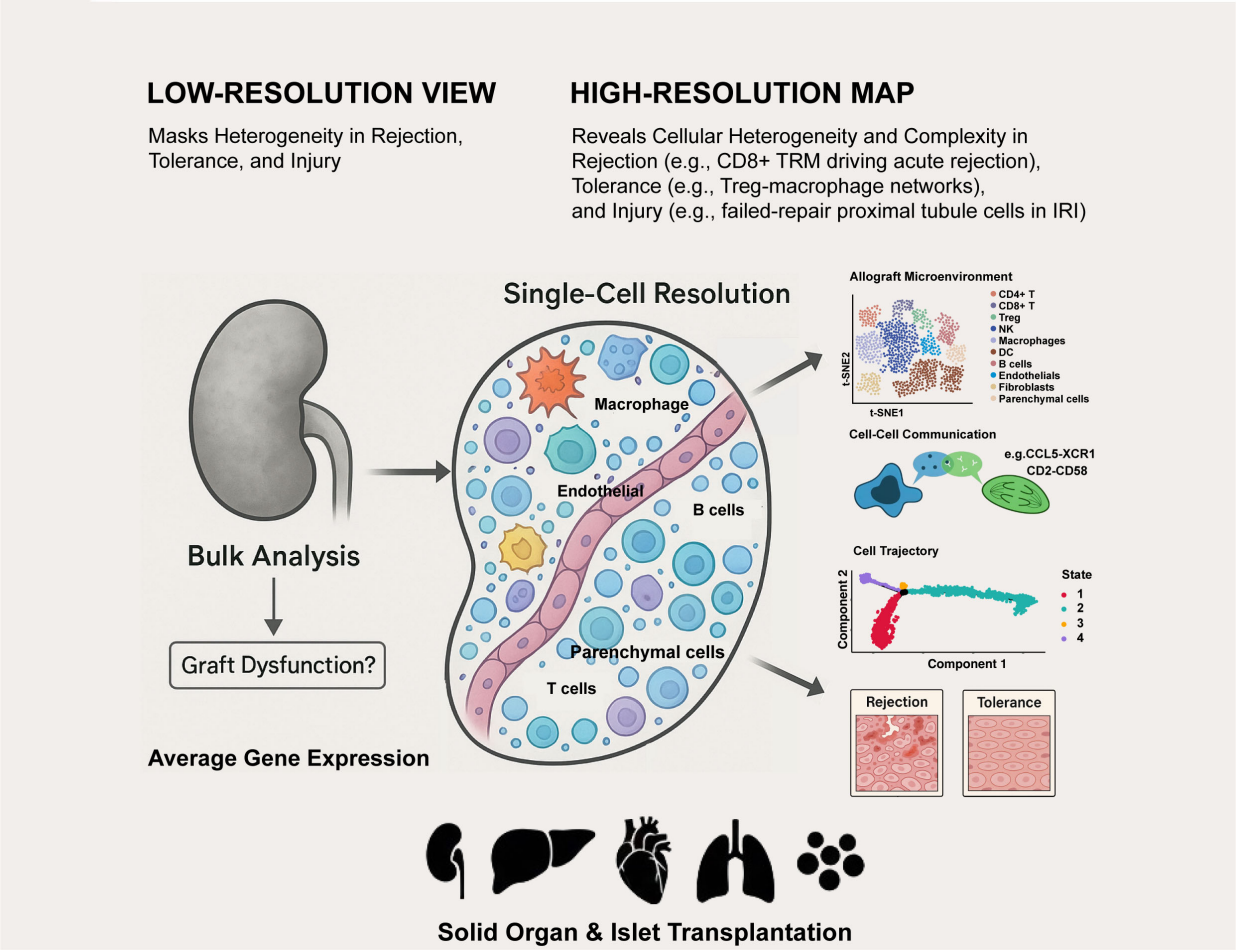
Single-cell technologies deconstruct the complex cellular landscape of transplanted organs.

Single-cell technologies have fundamentally changed our understanding of transplantation biology. It has taken us from a low-resolution, static perspective to a high-definition, dynamic map of the allograft microenvironment, revealing unprecedented and profound cellular heterogeneity and complexity in rejection, tolerance, and injury (**Figure 1**). Currently, the field is moving from simply identifying and classifying cell types to elucidating the causal mechanisms that lead to graft failure. By integrating multi-omics and spatial data, we are beginning to understand the gene regulatory networks, protein interactions, and tissue structures that determine graft fate (77).

The ultimate promise of these technologies lies in their clinical translation (108). The future of transplant medicine will involve combining single-cell data from non-invasive liquid biopsies with clinical and histological information. This will usher in a new era of precision medicine, characterized by (1) Predictive Diagnostics: Identifying high-risk patients before clinical symptoms of rejection appear; (2) Personalized Therapy: Tailoring immunosuppressive regimens based on an individual’s unique molecular rejection signature; (3) Innovative Therapeutics: Developing novel drugs that target the specific cell states and molecular pathways driving graft injury.

Although significant challenges remain, the rapid development of technology and computational methods gives us reason to believe that within the next decade, single-cell analysis will become an indispensable tool for the transplant clinician, leading us into a new era of higher graft survival rates and better quality of life for recipients.
